# The Role of Mast Cells and Their Inflammatory Mediators in Immunity

**DOI:** 10.3390/ijms241512130

**Published:** 2023-07-28

**Authors:** Theoharis C. Theoharides

**Affiliations:** 1 Institute for Neuro-Immune Medicine, Dr. Kiran C. Patel College of Osteopathic Medicine, Nova Southeastern University, Fort Lauderdale, FL 33328, USA; ttheohar@nova.edu; 2 Laboratory of Molecular Immunopharmacology and Drug Discovery, Department of Immunology, Tufts University School of Medicine, Boston, MA 02111, USA

Mast cells have existed for almost 500 million years [1] in many different species, including invertebrates and fish, in which they regulate inflammatory responses [2]. In mammals, mast cells are found in all tissues and express numerous surface receptors, allowing them to sense and respond to allergic, autoimmune, environmental, neurohormonal, pathogenic, and stress triggers by secreting numerous biologically active mediators. Of these mediators, secretory granule-stored histamine and tryptase are only the tip of the iceberg [3]. Consequently, mast cells could serve a critical role acting as pluripotent “immunoendocrine master players” [4]. However, a number of questions remain unanswered, such as questions involving their tissue characteristics and reactivity, especially in processes and conditions that do not involve allergic reactions.

The international Journal of Molecular Sciences recently published Special Issues dedicated to aspects of the pathophysiology of mast cells, such as “Mast cells in human health and diseases” edited by Giovanna Traina [5], and “Mast cells: when the best defense is an attack” edited by Margarita Martin [6], but these volumes focused on the role of mast cells in diseases, especially infections.

The emphasis of this Special Issue entitled “The Role of Mast Cells and Their Inflammatory Mediators in Immunity” was to address aspects of mast cell involvement in immune and inflammatory processes not covered by previous volumes.

A major review communicated via Dr. Domenico Ribatti and authored by Solimando et al. described in detail the production of interleukins by mast cells, as well as their important role in immune processes [7]. Given the proper microenvironment, such as one involving presence of IL-33, mast cells can release large amounts of chemokines CCL2 and CCL5 [8], as well as cytokines such as IL-1b [9] and TNF [10].

For the longest time, the notion persisted that all mast cells are the same. Then, they were separated into mucosal-type (MMC), containing only tryptase, and those of the connective-tissue-type (CTMC), containing both chymase and tryptase. However, increasing evidence indicates that there are unique subpopulations with distinct histological and secretory characteristics. The submission via Dr. Lars Hellman by Akula et al. using quantitative transcriptome analysis describes a particular subtype of mucosal mast cell in bronchoalveolar lavage fluid (BALF) that contributes to inflammation in the lungs of asthmatic horses [11] that may be applicable to humans.

The paper submitted via Dr. Claudia González-Espinosa by Martínez-Aguilar et al. identifies the augmenting effect of lysophosphatidylinositol (LPI, lysoPI) on mast cell functions promoting inflammation through the differential participation of the GPR55 and cannabinoid 2 (CB2) receptors [12]. PI serves as an endogenous lysophospholipid and endocannabinoid neurotransmitter, and is considered to be the endogenous ligand of GPR55. Utilizing murine-bone-marrow-derived mast cells (BMMCs), the authors found that LPI did not cause degranulation, but slightly increased FcεRI-dependent β-hexosaminidase release; however, LPI induced strong chemotaxis dependent on GPR55 receptor activation and movement towards tumors. LPI also induced the expression of IL-1β, TNF, and VEGF; however, in contrast to the chemotaxis, the effects on cytokine transcription were dependent on both GPR55 CB2 receptors [12].

The submission via Dr. Zenke by De Toledo et al. describes the important finding that human-induced pluripotent stem cell (hiPSC)-derived mast cells can mimic mastocytosis-like mast cells [13], suggesting that such cells may be used as a disease surrogate, thus abrogating the common use of immortalized mast cells (e.g., RBL, HMC-1, LAD2, and LADR) that do not accurately reflect normal mast cell functions.

The article by Drs. Tsilioni and Theoharides reports for the first time that nanomolar amounts of recombinant coronavirus spike protein stimulated human mast cells to secrete proteases via the activation of the angiotensin-converting enzyme 2 (ACE2) receptor, but stimulates the release of the proinflammatory cytokine IL-1b via the activation of TLR4; these processes are augmented by alarmin IL-33 [14]. This novel finding suggests that the spike protein may be able to stimulate mast cells via two different receptors, potentially utilizing different signal–transduction pathways. Moreover, the findings suggest that vaccines designed to stimulate antibody production against the ACE2 coronavirus receptor binding domain (RBD) may not protect against COVID-associated cytokine storms. As a result, mast cells could be involved in COVID-19 and Long-COVID syndrome via the release of different mediators [15].

The contribution communicated via Dr. Tsilioni by Jingshu et al. reported the development of a 3-D hydrogel formed by collagen I and culture human mast cells and showed that different Aβ peptides could stimulate mast cells to secrete inflammatory mediators. This model could incorporate microglia and neurons that could better reflect the mechanisms involved in the pathogenesis of neuroinflammation and Alzheimer’s disease [16].

The submission submitted via Dr. Marcus Maurer by Dr. Buttgereit et al. describes that the mitochondrial Complex I can act as a critical innate inhibitory component of the mast cell secretion of pro-inflammatory mediators [17]. Complex I (NADH:ubiquinone oxidoreductase) is the entry point for most electrons into the mitochondrial respiratory chain that eventually generates energy through oxidative phosphorylation in the form of ATP. While the inhibition of Complex I inhibited the primary human skin mast cell release of L-4, IL-5, IL-6, IL-13, TNF-α, and GM-CSF (but not release of IL-4 and IL-5) without loss of cell viability, Complex III did not. Instead, Complex III was reported to suppress regulatory T cells (Treg) since the Treg cell-specific ablation of Complex III in mice resulted in the development of fatal inflammatory disease, without affecting Treg cell number [18]. These results imply that mitochondrial respiratory Complexes may have affected mast cell function via mechanisms other than just energy metabolism.

Finally, the review by Theoharides and Kempuraj discusses the potential role of the ezrin, radixin, moesin (ERMS) family of proteins, especially moesin, in regulating the exocytotic mechanism of mast cell secretion of granule-stored mediators [19].

These papers highlight key aspects of the potential role of mast cells in immunity and inflammation by presenting novel findings on the subtypes, regulatory mechanisms, and unique associations with innate and external triggers. However, there are still gaps in our knowledge, such as the effect of the microenvironment on the tissue mast cell phenotype and ways to regulate mast cell activation. An improved understanding may be obtained through the use of in vitro disease surrogate models using organoids [20] with hiPSC-derived mast cells co-cultured with other relevant tissue cells, such as dendritic cells, endothelial cells, microglial cells, neurons, and pathogens. Such organoids could be used to investigate complex cell interactions, to study signal–transaction mechanisms, to identify new biomarkers of mast cell activation, and to screen for novel inhibitors.
